# Draft Whole-Genome Sequences of Three Shiga Toxin-Producing *Escherichia coli* O91:H21 Isolates, Two from Hemolytic Uremic Syndrome Patients and One of Porcine Origin

**DOI:** 10.1128/genomeA.01000-14

**Published:** 2014-10-09

**Authors:** Cindy H. Wu, Peng Zhang, Christina Q. Morales, David Kiang

**Affiliations:** California Department of Public Health, Food and Drug Laboratory Branch, Richmond, California, USA

## Abstract

This study presents three genomes of O91:H21 isolates, two from hemolytic uremic syndrome patients and one of porcine origin. Genome analyses reveal that one of the human isolates contains both Shiga toxin-encoding genes (*stx*_1_ and *stx*_2_), and all three isolates contain putative adhesin (*iha* and *eaeH*) and antibiotic resistance (*ampC*) genes.

## GENOME ANNOUNCEMENT

Non-O157 Shiga-toxin-producing *E. coli* (STEC) species are important emerging pathogens due to the rise of STEC-related illnesses worldwide (1). In 2011, an outbreak involving STEC O104:H4 led to 3,801 reported cases, and 46 deaths in Germany (2). Next-generation sequencing revealed STEC O104:H4 to be an enteroaggregative *E. coli* that acquired a Shiga-toxin gene associated with enterohemorrhagic *E. coli* strains (2, 3). The Germany outbreak illustrated that whole genome characterization is paramount for improving our understanding of genetic transfer among STEC strains and may lead to shortened response times.

STEC O91:H21 may progress to hemolytic uremic syndrome (HUS) in humans and has been implicated in outbreaks (4). Previous gene-specific characterizations revealed insights into its pathology and virulence (5–11). However, without whole-genome characterization, its pathogenic mode of action remains unclear. We obtained three O91:H21 isolates from the Michigan State University STEC Library Collection: two HUS patient isolates (H1 and H2) and one pork isolate (P). Whole-genome sequencing was performed using an Ion PGM (Thermo Fisher Scientific, Waltham, MA). Comparative analysis will be used to elucidate genomic differences among the isolates and potential influences on pathogenicity.

Genomic DNA was extracted from overnight culture using a DNAeasy blood and tissue kit (Qiagen, Valencia, CA). The genomes were sequenced on the Ion PGM (Thermofisher), and MIRA (v4.0.5) (12) was used for *de novo* assembly of the contigs. The assembly results for each isolate are as follows: H1, 292 contigs, average coverage of 23×, contig *N*_50_ of 40,255 bp, and genome size of 5,068,406 bp; H2, 133 contigs, average coverage of 32×, contig *N*_50_ of 140,146 bp, and genome size of 5,039,473 bp; P, 99 contigs, average coverage of 36×, contig *N*_50_ of 166,484 bp, and genome size of 4,982,843 bp. The G+C contents for isolates range from 46.6% to 51.5%.

The contigs were annotated using the CLC Genomic WorkBench Genome Finishing Module 1.3 (Qiagen, Boston, MA). H1 contains Shiga-toxin encoding genes, *stx*_1_ and *stx*_2_, whereas H2 and P only have *stx*_2_. H1 and H2 contain hemolysin gene, *hlyA*. All three isolates contain the antibiotic resistance gene, *ampC*. It is widely documented that this serogroup does not harbor intimin (*eae*) (13–16), and that other genes may contribute to attachment and effacing of host cells. Interestingly, our analysis reveals the presence of putative adhesin/intimin genes, *iha* and *eaeH*, in all three isolates. GenBank BLAST alignment indicates that the O91:H21 *eaeH* gene has 99.4% identity similarity to the German outbreak strain O104:H4 (CP003289) and 98.1% similarity to an enterotoxigenic *E. coli* strain E24377A (CU928145). Comparative analysis of whole genome sequences from these three isolates could provide insights into attributes of potentially re-emerging STEC strains and further reveal the influence of genomic differences on virulence. Observations from the comparison will be the focus of a future publication.

### Nucleotide sequence accession numbers.

GenBank accession numbers for the sequences are JNNI00000000 (H1), JNNJ00000000 (H2), and JNNL00000000 (P).
